# Effectiveness of Additional Structured Strength Training of Unaffected Lower Extremity on Balance and Gait Among Acute Poststroke Individuals

**DOI:** 10.1155/tswj/1663116

**Published:** 2025-04-02

**Authors:** Neha Kumari, Akshatha Nayak, Abraham M. Joshua, Shivananda D. Pai, Shyam Krishnan Krishna Kumar, Rinita Mascarenhas, Shreekanth D. Karnad

**Affiliations:** ^1^Department of Physiotherapy, Kasturba Medical College Mangalore, Manipal Academy of Higher Education, Karnataka, Manipal, India; ^2^Department of Neurology, Kasturba Medical College Mangalore, Manipal Academy of Higher Education, Karnataka, Manipal, India; ^3^Department of Neurology, Christian Medical College, Ludhiana, India

**Keywords:** balance, CVA, gait, muscle strength, resistance training, stroke, unaffected lower extremity

## Abstract

**Introduction:** Stroke reduces lower extremity muscle strength bilaterally, predominantly on the affected side. Stroke rehabilitation focuses on training the hemiparetic extremities, whereas functional activities require the recruitment of bilateral lower extremity muscles.

**Objectives:** This research is aimed at studying the effectiveness of additional structured strength training of unaffected lower extremity (ULE) on balance and gait among acute poststroke individuals.

**Methods:** This Nonrandomized Controlled Trial included 28 clinically stable acute poststroke individuals aged 20–80 years, with the first episode of stroke, and who could walk 5 m with or without assistive devices. The subjects were assigned to either an experimental group (*n* = 14) or a control group (*n* = 14). Both groups received 12 sessions of conventional stroke rehabilitation focusing on the affected side. In addition, individuals in the experimental group received structured strength training for the ULE.

**Main Outcome Measure:** Balance, gait, and muscle strength of the ULE were measured pre and after 2 weeks of intervention using Brunel Balance Assessment (BBA), Wisconsin Gait Scale (WGS), 2D gait analysis (Kinovea software), and a handheld dynamometer, respectively.

**Results:** The strength in the ULE of the experimental group improved significantly in all the muscle groups, whereas the control group showed improvements only in hip flexors, hip extensors, knee flexors, and ankle dorsiflexors. However, the strength gains in the hip flexors, hip abductors, knee extensors, and ankle dorsiflexors were significantly greater in the experimental group. Additionally, there was a significant difference among the groups in the BBA (*p* = 0.001) and WGS scores (*p* = 0.012). The kinematic variables of gait showed better knee flexion (*p* = 0.006), dorsiflexion angles (*p* = 0.016), and gait speed (*p* = 0.008) in the experimental group.

**Conclusion:** Additional structured lower extremity strengthening of the ULE led to improved strength of ULE, resulting in better balance function and gait among individuals with acute stroke.

**Trial Registration:** ClinicalTrials.gov identifier: CTRI/2018/12/016685

## 1. Introduction

Poststroke hemiplegia is prevalent in 70%–85% of first-stroke occurrences, with diminished muscle strength regarded as a significant factor in motor disability [1]. Hemiplegic patients typically demonstrate an asymmetric gait pattern and reduced gait velocity [2]. Among stroke survivors who initially experience walking impairment, 75% will eventually regain the ability to walk within 3 months [3]. This could lead to an increased risk of falls and greater dependency on caregivers until the individuals can walk independently [4–6].

To enhance poststroke recovery on gait and balance, evidence on rehabilitation focuses on a highly integrative and eclectic rehabilitation process, including mobility goals, passive facilitation of isolated movements, strengthening exercises, exercises on stationary bicycles or treadmills, impairment-oriented training, and robotics. Yet these approaches highlight the training of the affected lower extremity [7–9].

Previous findings reveal that a few affected descending corticospinal tract fibers remain ipsilateral, causing nonaffected extremity weakness [10–13]. Additionally, literature has stated that functional activities entail the recruitment of muscles in the bilateral lower extremity, suggesting strengthening the nonaffected extremity to facilitate improved recovery in poststroke individuals [14].

It is known that control of the affected upper extremity (UE) can be facilitated through symmetrical movements of bilateral extremities. These movements may allow the activation of the unaffected hemisphere, resulting in incremental activation of the damaged hemisphere that will improve the affected UE [15, 16]. It promotes neural plasticity by recruiting ipsilateral pathways from the uninvolved hemisphere to supplement the remaining crossed corticospinal pathways from the involved hemisphere, causing motor cortex disinhibition and upregulation of descending premotor neurons [17]. Based on the above principle, Jeon and Hwang trained the unaffected lower extremity (ULE) in addition to the affected extremity, improving dynamic balance and gait velocity among chronic poststroke individuals [18].

Strength loss in the unaffected side begins as early as 5 days poststroke [19]. Yet the research on ULE strengthening is limited; additionally, no standardized strengthening protocol exists for the ULE. Furthermore, studies on ULE strengthening exhibit ambiguity concerning its effects on enhancing postural control and gait parameters. This gap in the research is the primary aim of our study, which will employ a specific resistance training program on the unaffected side, alongside approach-oriented targeted training on the affected side.

## 2. Materials and Methods

### 2.1. Design

This study is a nonrandomized controlled trial.

### 2.2. Participants

The study was conducted at the Department of Physiotherapy, Kasturba Medical College (Manipal Academy of Higher Education), Mangalore, India, from December 2018 to January 2019. The Institutional Ethics Committee of Kasturba Medical College, Mangalore, approved the study (IEC KMC MLR 11–18/430).

**Figure 1:**
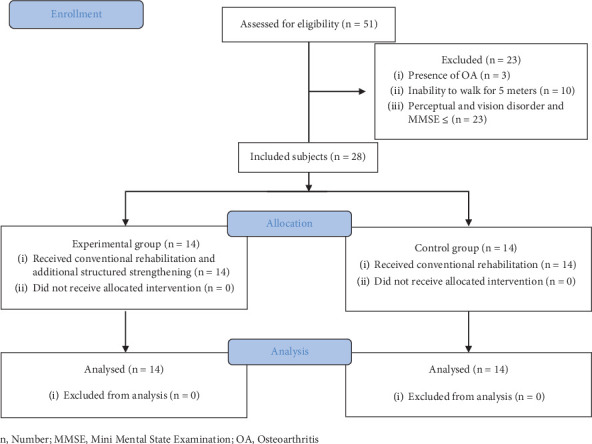
Participation flow chart. *n*, number; MMSE, Mini-Mental State Examination; OA, osteoarthritis.

The individuals diagnosed with stroke referred to the physiotherapy department for further rehabilitation were approached and explained about the study. A written informed consent was obtained, and they were screened for inclusion and exclusion criteria. Clinically stable individuals having a single episode of stroke diagnosed by a neurologist or a physician, aged 20–80 years, with an ability to follow commands (MMSE ≥ 23) (An unauthorized version of the MMSE was used by the study team without permission; however, this has now been rectified with PAR. The MMSE is a copyrighted instrument and may not be used or reproduced in whole or in part, in any form or language, or by any means without written permission of PAR (http://www.parinc.com/)). And the ability to walk 5 m with or without assistive devices was included in the study, whereas those with concurrent neurological conditions, cardiopulmonary, and musculoskeletal conditions were excluded from the study. If any individual had pushers syndrome or other perceptual or severe visual dysfunction, they were also excluded from the study. Demographic details and baseline data were collected postallocation. The procedure has been depicted in Figure 1, indicating the selection and allocation of the participants.

### 2.3. Intervention

The experimental and control group received conventional stroke rehabilitation for 2 weeks (12 sessions), with each session lasting for 45–60 min, including rest periods. The conventional stroke rehabilitation included facilitation and training of affected upper and lower extremities, functional re-education, balance, and mobility training in sitting and standing, along with gait training. The experimental group received additional strengthening exercises for ULE for 30 min, including rest periods, with rest periods not exceeding 90 s between the sets (Table 1). The protocol included the strengthening of flexors, abductors, extensors muscle groups of the hip, flexors and extensors of the knee, and dorsiflexion and plantar flexors groups of the ankle using standard strengthening techniques of three sets with 15 repetitions in each set. For each of the hip and knee muscles of ULE 1, RM was estimated using Epley's equation. The resistance applied was 60%–70% of 1 repetitive maximum for training. The repetitive maximum was reevaluated at the end of each week to accommodate for the improvement in strength.

**Figure 2:**
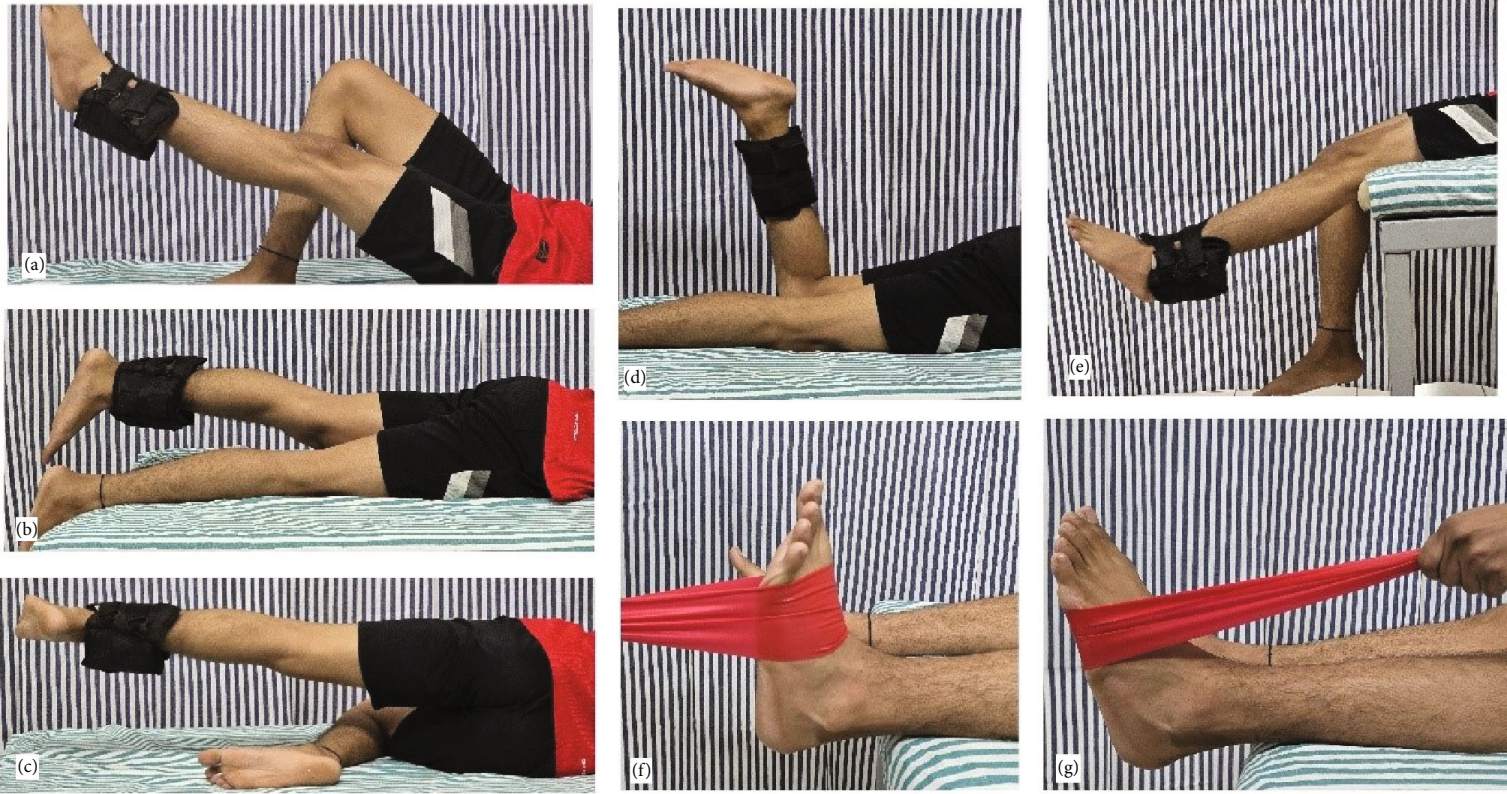
Strengthening exercises for the unaffected lower extremity. (a) Hip flexors. (b) Hip extensors. (c) Hip abductors. (d) Knee flexors. (e) Knee extensors. (f) Ankle dorsiflexors. (g) Ankle plantar flexors.

The strengthening of hip and knee muscles was done using free weights (weight cuffs) attached to the ankle, and the patient was positioned in a manner to facilitate unobstructed full-range movements against gravity. The patient was positioned in a supine for hip flexors, prone for hip extensors, and side-lying for hip abductors. Knee extensor strengthening was performed with the patient in high sitting, and knee flexors were strengthened with the patient in prone. For the exercises of the hip and knee muscles, the weight cuffs were strapped at the ankle. For exercising the ankle plantar and dorsiflexor, the Thera Band was looped around the forefoot, and the free end was held firmly by the therapist (Figure 2). The ankle dorsiflexors and plantar flexors were strengthened with the patient in a supine position with the foot out of the treatment couch. Three sets of 15 repetitions of each movement were given without varying the weights, interspersed with rest periods between sets. For ankle dorsiflexors and plantar flexors, the subject was asked to make a force against the therapist's resistance, and this was used to estimate approximate elastic resistance, which could be used to train these muscle groups. A suitable elastic band was chosen based on the manual testing results used in the standard positions. One repetitive maximum for hip and knee and the maximum voluntary contraction for ankle plantar and dorsiflexion were re-estimated at the end of each week to ensure the progression of resistance.

### 2.4. Assessment

The outcomes were measured pre and postintervention by an independent blinded assessor (physiotherapist) who was well-trained to perform the assessment. The outcome measures included muscle strength using a handheld dynamometer by baseline push–pull dynamometer. Functional balance and qualitative gait evaluation were assessed using the Brunel Balance Assessment (BBA) and the Wisconsin Gait Scale (WGS), respectively. In addition, a quantitative gait evaluation was performed using a 2D video graphic analysis using Kinovea software. The 2D video graphic gait analysis was performed based on the video recorded with the subjects were made to walk over a 5-m marked distance, using duct tapes with reflective markers applied on the subject's greater trochanter, lateral condyle of femur, lateral malleolus of the fibula, and head of the fifth metatarsal on the affected lower extremity. The video was recorded using a GoPro Hero5 HD camera (resolution of 4000 × 3000 pixels and a frame rate of 60 frames/second), mounted on a tripod with a spirit level, and kept at the midpoint of the walkway at a 4-m perpendicular distance with height corresponding to patient's hip. The angles of hip flexion, knee flexion, ankle dorsiflexion, and the time taken to complete a walking distance of 5 meters were calculated based on the recorded video using a freeware motion analysis software, Kinovea (Version 0.8.15).

Functional balance was measured with the BBA. BBA is a 12-point scale. Interitem correlations were < 0.9, coefficients of reproducibility and scalability were 0.99 and 0.69, respectively, and Cronbach's alpha was 0.92. Reliability was high (100% agreement) for both aspects of reliability. Correlations with other balance measures were significant (0.83/0.97, *p* < 0.01), indicating validity as a measure of balance disability [20]. The WGS has an interrater reliability of Cronbach's alpha coefficient of 0.85–0.88. The WGS has moderate construct validity with walking, balance, and functionality scales in patients with stroke. Further, the construct validity of the WGS was excellent with the FAC (*r* = −878), the BBS (*r* = −0.882), the PASS (*r* = −0.847), and the BI (*r* = −0.813) in subacute stroke [21].

### 2.5. Statistical Analysis

Data was entered and analyzed using a Statistical Package for the Social Science (SPSS) Version 25.0. The sample size was estimated using the formula **n** = (**Z**_(1 − **α**/2)_ + **Z**_**β**_)^2^ 2**σ**^2^/**d**^2^, where *Z*_*α*_ = 2.57 (for 1% *α* error), *Z*_*β*_ = 1.28 (90% power), *σ* (population variance) = 0.135 (derived from Berg balance scores reported in Table 2 of Jeon and Hwang [18]), and *d* (minimum detectable difference between the means) = 0.2 units. The calculated sample size was 28, 14 in the experimental and 14 in the control group.

Categorical variables in baseline data were compared across the experimental and control groups using the chi-square test, whereas quantitative variables were compared using an independent sample *t*-test. Within the group, the change in strength was compared using students' paired sample *t*-test, whereas the change in strength across groups postintervention was compared using an independent sample *t*-test. Pre and postintervention changes in BBA and WGS were analyzed using Wilcoxon's sign rank test, whereas the pre and postintervention change scores of the same between groups were compared using Mann–Whitney's *U* test. The within-group changes in angles of hip flexion, knee flexion, and ankle dorsiflexion pre and postintervention were compared using a paired sample *t*-test, whereas the pre–post difference in ROM across the group was analyzed using the independent sample *t*-test. Effect size was calculated by finding the mean difference between the groups and dividing by the pooled standard deviation. An intention-to-treat analysis was performed for the subjects lost to follow-up. A *p* value < 0.05 was considered statistically significant.

## 3. Results

The study included 28 participants allocated to the experimental group (*n* = 14) and control group (*n* = 14). Among the included subjects, 6 dropped out from the study because of distance from the rehabilitation center (*n* = 5) and the second episode of stroke (*n* = 1); hence, an intention to treat analysis was performed. The demographic details are depicted in Table 3 and reveal no statistically significant difference between the groups (*p* > 0.05) (Table 2).

Within the group, muscle strength changes in the ULE revealed a significant increase in strength for all the muscles tested among the experimental group; in contrast, among the control group, the improvement in the strength was noted only in hip flexors, hip extensors, knee flexors, and ankle plantar flexors. Compared to the control group, the experimental group showed significant strength gains in hip flexors, hip abductors, knee extensors, and ankle dorsiflexion (Table 3). Analysis of the effect size shows that hip flexors, hip extensors, hip abductors, knee extensors, and ankle dorsiflexors had large effect sizes, whereas knee flexors and ankle plantar flexors did not.

Within-group comparison of functional balance assessed using the BBA and the gait quality using the WGS revealed a statistically significant change in the experimental and control groups after 2 weeks of intervention; however, the experimental group showed better balance improvement than the control (Table 4).

Among the kinematic variables assessed in the present study after 2 weeks of intervention, the poststroke individuals in the control group did not show a significant change in hip flexion, knee flexion, and ankle dorsiflexion; in contrast, knee flexion and ankle dorsiflexion angles showed statistically significant differences among the experimental group with no significant change for hip flexion. When change scores were analyzed between the groups, only the knee flexion component showed a significant difference in the experimental group over the control group, and the effect size for the same was large. Gait speed improved both in the experimental and control groups after the intervention; however, the subjects in the experimental group improved with a large effect size and was significantly better change over the control group (Table 5).

## 4. Discussion

This study is aimed at assessing the impact of systematic ULE strength training on balance and gait kinematics variables using a standardized protocol in conjunction with conventional stroke rehabilitation.

The experimental group displayed increased muscle strength across all muscle groups, whereas the control group exhibited improved strength in specific muscle groups. The observed treatment effect was large for hip flexors, extensors, abductors, knee extensors, and ankle plantar flexors. The improvement among the experimental group following a structured strengthening program is in accordance with previous studies, where the muscle strength on the ULE improved following early mobilization and resistance exercises [18, 22]. The previous exercise protocols were designed to target the knee and ankle muscles of the ULE, while proprioceptive neuromuscular facilitation (PNF) was also employed bilaterally as a component of the ULE training regimen [18, 23–25]. The strategies used in the study demonstrated improvements in gait and balance. This observation could be attributed to the facilitation of early neural adaptations, which appear more pronounced than the adaptations typically seen with traditional resistance training. Indicating the mechanisms underlying these strategies may prioritize neural adaptation processes that enhance motor control and stability more effectively than resistance-based interventions [25]. Further, the improvement observed in the control group could be attributed to incorporating mobility, balance, and strengthening the ULE, which involves bilateral lower extremity engagement, thereby augmenting neural drive. Consequently, this increases intrinsic contractile strength, thereby facilitating muscle strength improvement [26].

The synchronized activity of both lower extremities plays a role in addressing overall postural instability and spatial neglect of posture [27–29]. Notably, deficits evident in the ULE and the affected lower extremity have been correlated with impaired balance and the emergence of gait asymmetry in the stroke-afflicted population [27]. Including exercises such as balance training, weight shifts, and reachouts in conventional rehabilitation is perhaps the possible reason for improved balance in both the experimental and control groups [30, 31]. However, the individuals in the experimental group showed considerable improvement in balance and gait due to muscle strength in hip flexors, hip abductors, knee extensors, and ankle plantar flexors. Contrary to most studies [22–25], our study observed an improvement in plantar flexor strength in the ULE of the control group. Ankle plantar flexors play a pivotal role in the stance phase of gait and contribute significantly to postural control [21, 32]. Nevertheless, it is essential to acknowledge the ULE muscle groups as they can influence postural control and gait [31, 32]. Our protocol for the control groups involved a more functional approach to affected lower extremity training, rather than targeting specific muscle groups. This functional approach may have contributed to increased muscle strength, potentially leading to improved gait speed in the control group.

The eclectic approach utilized to address gait impairments within conventional rehabilitation likely resulted in improvements in the spatial and temporal parameters of gait, as assessed using the WGS, in both the experimental and control groups. The considerable increase in the muscle strength in the ULE and better knee and ankle ranges seen in the experimental group would have influenced the gait performance, as it was previously found that unaffected side ankle dorsiflexors contributed to an enhanced gait speed in poststroke individuals [33, 34].

The improved knee and ankle control and improved strength of the ULE would have enhanced the gait speed in the experimental group. The result of the current study is consistent with a previous study by Sen et al. on the bilateral isokinetic strengthening of knee and ankle muscle groups, which led to an improvement in balance, gait performance, and mobility index among poststroke individuals [25]. Gait speed, among all parameters, is the best indicator of the efficacy of ambulatory function in subjects with stroke [35]. The improvement in gait speed with a significantly large effect size observed in the experimental group can be attributed to the intervention given, and hence, strengthening of ULE could be recommended as a part of the standard intervention in the management of gait pathologies in stroke.

Although our study has highlighted an effective protocol for improving balance and gait poststroke, the present study has limitations and can be added or studied separately. Firstly, there is an absence of long-term follow-up to assess the retention effect of the strength training on the functional gains. Secondly, if assessed, the strength gains of the affected lower extremity could give a better insight into the findings. Thirdly, six individuals in the control group had dropped out. Future studies could be done with a larger sample size and a long-term treatment duration, and follow-up is required to generalize the study results. Furthermore, the assessment could include a 3D gait analysis and electromyography to assess the gait parameters and the recruitment of lower extremity muscles. Additionally, categorization based on the duration of the stroke and its effect on bilateral training of the lower extremity could be compared and studied in the future.

## 5. Conclusion

The study findings revealed improved strength in the ULE following a structured strengthening program, which led to more significant improvement in functional balance and spatiotemporal gait parameters. Hence, these findings suggest that structured strength training of ULE should be incorporated into conventional poststroke rehabilitation to improve balance and gait among acute poststroke individuals.

## Figures and Tables

**Table 1:** Comparison of rehabilitation protocols: control versus experimental groups.

**Control**	**Experimental**
• Duration: 45–60 min • Conventional rehab • Treatment: facilitation and training of paretic upper and lower extremities, functional re-education, balance, and mobility training in sitting and standing, along with gait training	• Duration: 75–90 min • Conventional rehab + strengthening for ULE • Treatment: additionally strengthening for hip flexors, hip extensors, knee flexors, knee extensors, ankle dorsiflexor, and ankle plantar flexors. • Dosage: 3 × 15 reps • Resistance: 60%–70% of 1 RM

**Table 2:** Baseline demographic data.

**Variables**		**Experimental group** **n** ** (%)**	**Control group** **n** ** (%)**	**p** ** value** ^**a**^
Age (years)	20–40	2 (14.3)	2 (14.3)	1.000
	40–60	4 (28.6)	8 (57.1)	
	60–80	8 (57.1)	4 (28.6)	
	Mean ± SD	52.14 ± 12.87	59.5 ± 15.76	0.188

Gender	Male	11 (78.6)	9 (64.3)	0.403
	Female	3 (21.4)	5 (35.7)	

Type of stroke	Ischemic	13 (98.9)	13 (98.9)	1.000
	Hemorrhagic	1 (7.1)	1 (7.1)	

Side of involvement	Right	8 (57.1)	8 (57.1)	1.000
	Left	6 (42.9)	6 (42.9)	

Poststroke duration (days)	01–10	12 (85.7)	11 (78.6)	1.000
	11–20	2 (14.3)	2 (14.3)	
	21–30	0 (0)	1 (7.1)	
	Mean ± SD	6.93 ± 5.17	5.57 ± 3.46	0.421

*Note: n*, number of subjects; *p* < 0.05, significant.

Abbreviations: IQR, interquartile range; SD, standard deviation.

^a^Chi-square test.

**Table 3:** Change scores of the muscle strength pre and postintervention.

**Muscle strength (kg)**	**Groups**	**Baseline ** **m** **e** **a** **n** ± **SD**	**2 weeks ** **m** **e** **a** **n** ± **SD**	**p** ** value (within-group)** ^**£**^	**Effect size (** **m** **e** **a** **n** ± **SD) (between group)**	**p** ** value (between group)** ^**€**^
Hip flexors	Experimental	4.14 ± 1.02	5.24 ± 1.25	< 0.001⁣^∗^	1.211 (0.79 ± 0.65)	0.005⁣^∗^
	Control	4.00 ± 1.03	4.28 ± 1.13	0.046⁣^∗^		

Hip extensors	Experimental	3.50 ± 1.50	4.28 ± 1.32	0.001⁣^∗^	0.589 (0.36 ± 0.61)	0.136
	Control	2.50 ± 1.22	2.92 ± 1.32	0.008⁣^∗^		

Hip abductors	Experimental	3.71 ± 0.91	4.78 ± 1.62	0.006⁣^∗^	0.96 (0.86 ± 0.89)	0.024⁣^∗^
	Control	3.14 ± 0.77	3.36 ± 0.92	0.189		

Knee flexors	Experimental	4.21 ± 1.25	4.71 ± 1.38	0.047⁣^∗^	0.00 (0 ± 0.81)	0.935
	Control	3.64 ± 0.92	4.14 ± 1.16	0.0029⁣^∗^		

Knee extensors	Experimental	4.21 ± 1.25	5.42 ± 1.08	0.002⁣^∗^	0.941 (0.86 ± 0.91)	0.025⁣^∗^
	Control	4.28 ± 1.20	4.64 ± 1.27	0.055		

Ankle plantar flexors	Experimental	2.85 ± 1.09	3.71 ± 1.13	0.0121⁣^∗^	0.365 (0.36 ± 0.98)	0.346
	Control	2.14 ± 0.66	2.64 ± 0.74	0.047⁣^∗^		

Ankle Dorsiflexors	Experimental	2.64 ± 0.84	4.00 ± 1.03	< 0.001⁣^∗^	1.576 (1.07 ± 0.68)	< 0.001⁣^∗^
	Control	2.35 ± 0.49	2.64 ± 0.74	0.165		

*Note:* Effect size interpretation ranges are < 0.1: trivial effect; 0.1–0.3: small effect; 0.3–0.5: moderate effect; > 0.5: large difference effect.

Abbreviation: IQR, interquartile range.

^£^Paired sample *t* test.

^€^Independent sample *t* test.

⁣^∗^Significant.

**Table 4:** Change scores of BBA and WGS scores.

**Variables**	**Groups**	**Baseline median (IQR)**	**2 weeks median (IQR)**	**p** ** value (within-group)** ^**£**^	**Median difference (between group)**	**p** ** value (between group)** ^**€**^
BBA	Experimental	6.50 (6, 8)	10 (9, 11)	0.001⁣^∗^	1.5	0.001⁣^∗^
	Control	8 (7, 9)	10 (7.75, 11)	0.016⁣^∗^		

WGS	Experimental	25.7 (22.4, 28.5)	16.2 (13.91, 20.53)	0.001⁣^∗^	3.42	0.012⁣^∗^
	Control	26.1 (24.32, 27.95)	20.02 (17.1, 26.95)	0.008⁣^∗^		

Abbreviations: BBA, Brunel Balance Assessment; IQR, interquartile range; WGS, Wisconsin Gait Scale.

^£^Wilcoxon's sign rank test.

^€^Mann–Whitney's *U* test.

⁣^∗^Significant.

**Table 5:** Change scores of kinematic gait parameters.

**Gait parameters**	**Groups**	**Baseline ** **m** **e** **a** **n** ± **SD**	**2 weeks ** **m** **e** **a** **n** ± **SD**	**p** ** value (within-group)** ^**€**^	**Effect size ** **m** **e** **a** **n** ± **SD (between group)**	**p** ** value (between group)** ^***δ***^
Hip flexion^0^	Experimental	16.28 ± 4.82	19.35 ± 4.95	0.066	0.761 (3.21 ± 4.19)	0.073
	Control	17.21 ± 4.45	17.07 ± 3.26	0.844		

Knee flexion^0^	Experimental	−1.28 ± 4.99	−5.35 ± 3.56	0.006⁣^∗^	0.841 (3.43 ± 4.08)	0.037⁣^∗^
	Control	1.21 ± −2.25	0.57 ± −3.25	0.507		

Ankle dorsiflexion^0^	Experimental	10.14 ± 4.34	7.42 ± 3.82	0.016⁣^∗^	0.361 (1.21 ± 3.36)	0.350
	Control	4.85 ± 3.93	6.35 ± 4.37	0.089		

Gait speed^&^	Experimental	22.14 ± 3.99	15.42 ± 4.66	< 0.001⁣^∗^	1.093 (4.21 ± 3.85)	0.008⁣^∗^
	Control	17.64 ± 3.91	15.14 ± 5.82	0.029⁣^∗^		

*Note:* Effect size interpretation ranges are < 0.1: trivial effect; 0.1–0.3: small effect; 0.3–0.5: moderate effect; > 0.5: large difference effect.

Abbreviation: SD, standard deviation.

^0^Degree.

^&^Gait speed in seconds.

^€^Paired sample *t*-test.

^*δ*^Independent sample *t*-test.

⁣^∗^Significant.

## Data Availability

The data that support the findings of this study are available from the corresponding author upon reasonable request.
